# Treatment of Fractures of Long Bones by Simple Extension

**Published:** 1861-10

**Authors:** 


Treatment of Fractures of Long Bones by Simple Extension. By John Swinburne, M. D., one of the Surgeons to the Albany City Hospital. Albany: Van Benthuysen, Printer, 1861.

This is a pamphlet edition of the sixteenth paper, in the last volume of the *Transactions of the State Medical Society of New York*—for a copy of which we are indebted to the courtesy of the author.

Dr. Swinburne's views have been before the profession during the past three years or more, having been first presented at the meeting of the above Society in 1859; but Dr. S. has personally tested his plan during some thirteen years, in private and hospital practice. We think his *brochure* valuable, in that it will serve to direct the minds of those who read it, to the fact that much of the present bandaging and dressing is entirely unnecessary; and, which is far more important, that

**London Lancet*, and *Dublin Quarterly Journal of Medical Science*,—reviews of Prof. S. D. Gross' *System of Surgery*, 1861.

it persistently presses home the great central fact in the correct treatment of fractures, viz: *the absolute necessity of securing proper extension and counter-extension.* But the plan of treatment is, by no means, so novel and revolutionary as one would be led to believe from its title, or the discussion it has occasioned.

Briefly, the modes of treatment proposed by Dr. Swinburne, are as follows: *In fractures of the femur*, the patient is placed in bed, and a broad, well-padded belt, passed beneath the perineum and over the groin of the injured side, is fastened to the head of the bedstead—furnishing the counter-extension; while extension is made by the use of adhesive strips, attached to the leg, and fastened, by a cord, to the foot of the bedstead. The position of the foot is controlled by a strip of cloth or plaster, or by a bran or sand bag. Forty cases have been treated in this manner, in but one of which "was there visible shortening, nor was there any distortion of the thigh; no eversion or inversion of the foot. The average period of time during which extension was maintained was five weeks; and in the majority of cases, union was tolerably firm at the expiration of from fifteen to thirty days, according to the age of the patient and the nature of the injury." Of these cases occurring at all parts of the shaft and neck, some were oblique, some compound, some comminuted, and in one case four inches of the bone was crushed in fragments. "Two were cases where the thigh and leg were *both* fractured, and in *all* the results may be considered *perfect;* for where shortening only amounts to half an inch, it can be ascertained only by actual measurement. Of these forty cases, twelve were fractures within the capsular ligament, occurring in patients, most of them over sixty years of age, and all treated by this method of extension, with results much better than could be expected, and which it would have been in vain to expect under the usual treatment."

*In fractures of the tibia and fibula*, extension and counter-extension are made "through the medium of a delicate splint, and an equally delicate foot-piece,"—to all intents and purposes, a Desault's splint; though, where the seat of fracture is near the knee-joint, the treatment is the same as in fractured femur. In compound fractures of both bones, the double-inclined plane or hinged splint, is preferred, to remedy the tendency of the superior fragment to anterior displacement; though other modes are used.

*In fractures of the upper extremities*, the principle involved is the same. In the humerus, simple extension is made by having a splint extending three inches below the elbow and the same distance above the shoulder. The elbow is secured to the lower part of the strip of board by means of adhesive plaster passed through an opening, while to its upper extremity it has fastened the extremities of an axillary belt. Lateral motion is to be prevented by the use of strips of plaster binding the arm to the splint. Fracture in or near the elbow-joint is treated by dressing the arm on a hinged-splint, whose superior extremity is furnished with an axillary belt, or is strapped to the arm with adhesive plaster. To the inferior extremity the hand is secured also by adhesive plaster, and sufficient extension and counter-extension is then obtained by flexing the arm at the elbow. The position of the hinge, above or below the elbow, regulates the seat of extension; if placed above, the extension is made between the elbow and hand, if below, between the elbow and the shoulder. Colles' fracture is treated by a splint extending from the point of the elbow to the metacarpo-phalangeal articulation, supplied with two compresses, corresponding to the concavities at the elbow and carpus.

We have not space for further detail, referring to the work itself for much that is well worthy of perusal; but merely add,—Dr. Swinburne's improvement deriving so much of its value from its total disuse of bandages,—the following remarks on this subject:

* * * There is no need of bandaging in the treatment of fractures at all, and the treatment is more speedy and comfortable to the patient without bandages than with them. I have now treated, without any bandages whatever, forty fractures of the thigh, in every portion of its shaft, simple, compound, comminuted, and intra-capsular, and with better results than would be obtained by splints and bandages. I

have treated over one hundred fractures of the long bones with no bandaging, and only dressing enough to perpetuate the extension, which, in my humble judgment, is much better than trying to force the bones to maintain their position by lateral pressure with bandages, compresses and splints.

If the source of irritation, (the fractured ends of the bones,) is removed by their reduction and maintainance by such means as will not obstruct circulation and cause infiltration and inflammation, the precautions as to bandaging are unnecessary. If bandaging, at the time of its application, does not produce the slightest constriction anywhere, the subsequent infiltration and inflammation of the tissues will soon make the limb an uncomfortable bedfellow, as every day's practice will sufficiently attest.

I think it is not the bandaging at the time that produces all the bad results we occasionally see; it is the subsequent swelling. I regret to say that the profession, sometimes, sacrifice everything to appearance. A beautiful dressing (with them) is synonymous with a good result.

Now, if there is no necessity of bandages in the treatment of fractures, why not dispense with them as much as possible, as they are painful and troublesome in their application, and in all the subsequent dressings still more so.

They constrict the limb, producing more or less mischief—1st, by the extra warmth of the parts; 2d, obstruction to circulation; 3d, difficulty of examining carefully the position of the bones; 4th, the trouble, pain and disturbance of the bones in re-dressings. Per contra, by the plan under discussion the limb is kept cool, is examined at will, and little constriction ensues. Hence, little or no pain is experienced during the process of reparation, and no disturbance caused by re-dressing.

